# Editorial: Exploring neuromodulation and neuroimaging techniques for psychiatric disorders: insights from preclinical and clinical studies

**DOI:** 10.3389/fnhum.2026.1790803

**Published:** 2026-01-30

**Authors:** Unal Sakoglu, Martin Dietz, Elisa Kallioniemi

**Affiliations:** 1 Department of Computer Engineering, University of Houston–Clear Lake, Houston, TX, United States; 2 Department of Clinical Medicine, Faculty of Health, Center for Functionally Integrative Neuroscience, Aarhus University, Aarhus, Denmark; 3 Department of Biomedical Engineering, New Jersey Institute of Technology, Newark, NJ, United States

**Keywords:** deep brain stimulation (DBS), functional near-infrared spectroscopy (fNIRS), neuroimaging, neuromodulation, psychiatric disorders, repetitive transcranial magnetic stimulation (rTMS), temporal interference stimulation, transcranial direct current stimulation (tDCS)

Over the past decade, rapidly advancing neuroimaging and neuromodulation technologies have transformed our understanding of healthy brain function and its dysfunction in disease (Fox et al., 2014; Fregni et al., 2021; Desarkar et al., 2024). In psychiatry, human neuroimaging methods have enabled precise mapping of neural circuits (Etkin, 2019). Meanwhile, neuromodulation techniques have provided tools to causally influence and alter targeted networks (Fitzgerald, 2011). The combination of these approaches can inform where and how to stimulate, and test whether the circuits identified through imaging actually drive symptoms and whether altering them produces clinical benefit (Kas et al., 2025).

This Research Topic brought together preclinical and clinical studies that leveraged neuroimaging and neuromodulation to advance diagnosis, treatment, and mechanistic insight across psychiatric disorders. In this Editorial, we summarize the key findings from the five contributing works. By highlighting these studies, we aim to elucidate the promise and challenges of neuromodulation and neuroimaging techniques in the study of psychiatric disorders.

In a rodent model, Lu et al. used deep brain stimulation (DBS) of the ventral tegmental area (VTA) to explore the neural mechanisms underlying positive psychotic-like behaviors. The results showed that stimulation reduced hyperlocomotion and stereotyped behaviors. Using local field potential recordings, fiber photometry, and optogenetics, the authors showed that local GABAergic neurons in the VTA, rather than dopaminergic neurons, were responsible for mediating these effects. This study contributed to the mechanistic understanding of circuit-specific effects of DBS and provides preliminary evidence that targeting the VTA may be beneficial in treating the positive symptoms of psychosis.

While preclinical DBS studies provide critical mechanistic insight into how modulation of specific brain circuits can influence symptoms and behavior, translating these findings to broader clinical populations requires stimulation approaches capable of achieving precise, focal modulation of deep brain structures. Yatsuda et al. examined temporal interference (TI) stimulation for targeting deep brain regions at the population level. They found that the same montage consistently modulated deep targets across populations, although with variable focality. The trade-off between deep stimulation and unwanted cortical modulation was target-dependent. Furthermore, compared to transcranial alternating current stimulation, TI reduced unwanted cortical modulation at least 1.5-fold, but showed higher variability. These findings offer practical guidance for montage selection while highlighting the target-dependent trade-offs that must be considered when applying TI clinically.

Targeting considerations also motivated the work of Wu et al., who presented a protocol to test whether repetitive transcranial magnetic stimulation (rTMS) targeting the dorsomedial prefrontal cortex can alleviate anhedonia in schizophrenia. Their trial will randomize 82 patients to active or sham stimulation using an intermittent theta-burst protocol twice daily for 15 days. Assessments will include anhedonia symptoms, cognitive function, and prefrontal hemodynamic activity via functional near-infrared spectroscopy (fNIRS) at baseline, post-treatment, and four-week follow-up. If the clinical trial proves effective, this approach could offer a new intervention strategy for negative symptoms.

fNIRS also featured in the work of Hui et al., although here as a diagnostic marker rather than the outcome measure. They compared prefrontal activation during verbal fluency tasks using fNIRS in individuals with bipolar disorder (BD), unipolar depression (UD), and healthy participants. The results indicated that both the BD and UD groups showed less oxygenated hemoglobin activation than controls, with BD showing the least total activation. Activation in the ventrolateral prefrontal cortex during a semantic fluency task differed between BD and UD, suggesting a potential biomarker for differential diagnosis. Moreover, the phonological fluency task produced greater prefrontal activation than the semantic fluency task for UD and control participants, but not for those with BD, indicating that patterns of neural engagement in these tasks may be different between these groups.

Brunelin et al. demonstrated circuit-informed neuromodulation in practice in a case study of hoarding disorder. This disorder is typically resistant to existing treatments. They targeted the right dorsolateral prefrontal cortex, which plays a role in impulse control. Accelerated cathodal high-definition transcranial direct current stimulation (tDCS) was applied with three 20-min daily sessions over 5 days. There were improvements in acquisition behavior and mood, while no adverse effects were observed. While a single case cannot establish efficacy, this report suggests that neuromodulation may offer new possibilities for people with limited therapeutic options.

Together, these five contributions illustrate how neuroimaging and neuromodulation, applied independently or in combination, can advance psychiatric research from multiple perspectives. As the technologies continue to mature, they will offer increasingly precise ways to understand, diagnose, and treat psychiatric disorders.
